# The Effect of Statin Added to Systemic Anticancer Therapy: A Meta-Analysis of Randomized, Controlled Trials

**DOI:** 10.3390/jcm7100325

**Published:** 2018-10-04

**Authors:** Hyun Joo Jang, Hyeong Su Kim, Jung Han Kim, Jin Lee

**Affiliations:** 1 Division of Gastroenterology, Department of Internal Medicine, Dongtan Sacred-Heart Hospital, Hallym University Medical Center, Hallym University College of Medicine, Hwasung 18450, Gyeonggi-Do, Korea; jinlee@hallym.or.kr; 2 Division of Hemato-Oncology, Department of Internal Medicine, Hallym University Medical Center, Hallym University College of Medicine, Seoul 07441, Korea; nep2n@hallym.or.kr

**Keywords:** HMG CoA reductase inhibitor, statin, cancer, randomized, meta-analysis, review

## Abstract

Preclinical studies have demonstrated that statins have anticancer properties and act in an additive or synergistic way when combined with anticancer therapy. We conducted this meta-analysis of randomized, controlled phase II or III trials to evaluate the effect of statins added to systemic anticancer therapy in patients with solid cancer. A systematic literature search was performed to identify all randomized trials that were designed to investigate the effect of statins in patients with cancer using PubMed, EMBASE, Google Scholar, and Web of Science (up to August 2018). From eight randomized controlled trials, 1760 patients were included in the pooled analyses of odds ratios (ORs) with 95% confidence intervals (CIs) for grade 3–5 adverse events (AEs) and overall response rate (ORR) and hazard ratios (HRs) with 95% CIs for progression-free survival (PFS) and overall survival (OS). The addition of statin to anticancer agents did not significantly increase the incidence of grade 3–5 AEs (OR = 1.03, 95% CI: 0.81–1.29, *p* = 0.78). However, the combination of statin and anticancer agents did not improve ORR (OR = 0.96, 95% CI: 0.77–1.20, *p* = 0.72) compared with that of anticancer therapy alone. In addition, statins added to systemic anticancer therapy failed to prolong PFS (HR = 0.99, 95% CI: 0.90–1.10, *p* = 0.92) and OS (HR = 0.91, 95% CI: 0.76–1.11, *p* = 0.52). In conclusion, this meta-analysis of randomized controlled trials does not support clinical benefits of statins added to systemic anticancer therapy in patients with solid cancer.

## 1. Introduction

Statins are inhibitors of the 3-hydroxy-3-methylglutaryl-coenzyme A (HMG-CoA) reductase, which is the rate-limiting enzyme in cholesterol formation. Numerous studies have demonstrated that statins can not only decrease serum total and low-density lipoprotein (LDL) cholesterol concentrations, but also reduce ischemic cardiovascular disease-related morbidity and mortality [1,2]. Thus, statins are inexpensive and effective agents for prevention and treatment of cardiovascular disease.

For more than a decade, however, there had been a scientific debate on the association between long-term use of statins and the risk of developing cancer. Since experimental studies suggested that statins may be carcinogenic [3], clinical trials observed a significant increased incidence of cancer in patients treated with statins [4,5]. However, other studies yielded different results that statins had no relationship with cancer risk [6,7,8]. Statins inhibit the rate-limiting step of the mevalonate pathway in which mevalonic acid is the precursor in the biosynthesis of isoprenoid molecules such as cholesterol, dolichol, and ubiquinone. Mevalonate-derived prenyl groups, farnesyl pyrophosphate (FPP) and geranylgeranyl pyrophosphate (GGPP), facilitate intracellular functions of various proteins [9,10]. FPP and GGPP are essential substrates for posttranslational modification of rat sarcoma viral oncogene homolog (RAS) and RAS homologue (RHO), which have important roles in growth, proliferation, migration, and survival [11]. Based on the role of statin on the posttranslational modifications of RAS and RHO, its potential antitumor effect has also been investigated [12,13,14,15,16]. Experimental studies have demonstrated that statins can inhibit tumor growth and induce apoptosis in a variety of cancer cell lines, including colorectal cancer (CRC) [12], pancreatic cancer (PC) [13], cholangiocarcinoma [14], breast cancer [15], and small-cell lung cancer (SCLC) [16]. In addition, many observational studies have reported that stains can reduce recurrence or mortality in various types of cancer [17,18,19,20,21,22].

Statins may act in additive or synergistic ways when combined with anticancer agents. Indeed, many preclinical studies have demonstrated the synergistic interaction of statins with chemotherapeutic agents, such as doxorubicin [15], paclitaxel [23], 5-fluorouracil (5-FU) [24], platinum [25], irinotecan [26], gemcitabine [27], and gefitinib [28]. In addition, a phase II study reported that simvastatin in combination with irinotecan/5-FU/leucovorin (FOLFIRI) chemotherapy was effective and feasible with no additive side-effects in patients with metastatic CRC [29].

Based on these observations, randomized phase II or III clinical trials have been conducted to investigate the effect of adding statins to anticancer treatment in various types of cancer [30,31,32,33,34,35,36,37]. Whereas the first randomized trial of pravastatin in patients with hepatocellular carcinoma (HCC) reported a 9-month increase of overall survival (OS) [30], other randomized, controlled trials failed to demonstrate a significant improvement of clinical outcomes in patients treated with statin in combination with anticancer agents [31,32,33,34,35,36,37]. However, the results were inconclusive because some studies had a small sample size with a lack of blinding. Therefore, we conducted this meta-analysis of randomized, controlled trials to evaluate the effect of statin added to systemic anticancer therapy.

## 2. Materials and Methods

### 2.1. Search Strategy

This meta-analysis was conducted according to the Preferred Reporting Items for Systematic Reviews and Meta-Analyses (PRISMA) guidelines [38,39]. A systematic literature search was performed to identify all randomized, controlled trials that were specifically designed to evaluate the effect of statins among patients with cancer using PubMed, EMBASE, Google Scholar, and Web of Science (up to August 2018). The following search terms were used: “hydroxymethylglutaryl coenzyme A reductase inhibitor” or “HMG-CoA reductase inhibitor” or “statin” or “pravastatin” or “simvastatin” or “fluvastatin” or “atorvastatin” or “rosuvastatin” or “lovastatin” AND “carcinoma” or “cancer” or “neoplasm” or “malignancy” AND “randomized.” All eligible studies were retrieved and their bibliographies were checked for other relevant publications. We also scanned the reference lists of the retrieved articles. We did not contact authors of the original studies for additional information.

### 2.2. Selection Criteria

Eligible studies met the following inclusion criteria: (i) randomized clinical trial in malignant solid tumors; (ii) randomization of patients to systemic anticancer therapy with or without a HMG-CoA reductase inhibitor (statin); (iii) sufficient data for odds ratio (OR) with 95% confidence interval (CI) for overall response rate (ORR) or grade 3–5 adverse events (AEs) and/or hazard ratio (HR) with 95% CI for progression-free survival (PFS) or OS; (iv) studies published only in peer-reviewed journals; and (v) articles written in English.

### 2.3. Data Extraction

Two investigators (H.J.J. and H.S.K.) independently screened relevant studies. They carefully checked the titles and abstracts from the initial search and excluded articles that did not meet the inclusion criteria. Then, the eligible articles were completely reviewed and the needed data were extracted. Any disagreement was resolved through discussion with the principle investigator (J.H.K.).

The following data were gathered from the included studies: first author, year of publication, trial phase, treatment setting and regimen, primary endpoint, number of patients, ORR, incidence of grade 3–5 AEs, and survival outcomes (PFS and/or OS) along with their HRs with 95% CIs. When both univariate and multivariate analysis were performed to get the HR for PFS or OS, the data from multivariate analysis were selected preferentially.

### 2.4. Statistical Analyses

Statistical values used in the analyses were directly extracted from the original articles. If HRs with their 95% CIs were not provided, the Engauge Digitizer software was used to estimate them from the Kaplan-Meier curves.

The RevMan version 5.3 (Cochrane Collaboration, Copenhagen, Denmark) was used to combine the data. The heterogeneity across studies was estimated by using the *I*^2^ inconsistency test and Cochran’s Q statistic test. The fixed-effect model (Mantel–Haenszel method) was selected if there was no substantial heterogeneity (*p* ≥ 0.1 or *I^2^* *≤* 50%). When significant heterogeneity was observed (*p* < 0.1 and *I^2^* > 50%), the random-effects model (DerSimonian-Laird method) was adopted. We planned to perform additional subgroup analyses to identify the source of heterogeneity. The plots show a summary estimate of the results from all studies pooled. The size of each square represents the estimate from each trial, reflecting its statistical weight. Outcomes are shown as forest plots with diamonds representing the estimate of the pooled effect. The width of each diamond indicates its precision. The line of no effect is number one for binary outcomes, which implies statistical significance if not crossed by the diamond [40]. Statistical significance of the pooled HR or OR was determined by the *Z*-test. The pooled OR < 1.0 and HR < 1.0 indicate higher rate and better survival, respectively, for the addition of statin to anticancer therapy.

Publication bias was assessed graphically by the Begg’s funnel plot and quantified by the Egger’s test [41,42]. Statistical significance was considered for a *p*-value of less than 0.05.

### 2.5. Quality of the Included Studies

The methodological quality of the randomized trials was scored using the Jadad five-item scale, taking into account randomization, double blinding process, and withdrawals or dropouts [43]. The final score ranged from 0 to 5, with high quality studies having a score of ≥3.

## 3. Results

### 3.1. Results of Search

**Figure 1:**
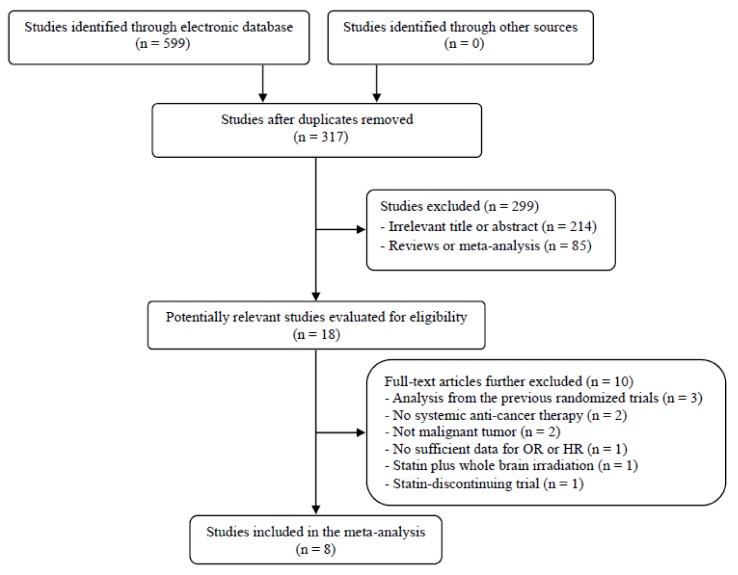
Flow diagram of search process.

The flow diagram of the search process is shown in Figure 1. A total of 317 potentially relevant articles were initially retrieved after removing duplicates, but 299 of them were excluded after careful screening of the titles and abstracts. Of the remaining 18 potentially eligible studies, 10 were further excluded by the inclusion criteria. Eventually, the eight randomized studies fulfilling the eligibility criteria were included in the meta-analysis [30,31,32,33,34,35,36,37].

### 3.2. Characteristics of the Included Studies

Table 1 summarizes the main characteristics and clinical outcomes of the eight eligible studies. Most studies were conducted in patients with advanced or metastatic cancer. The studies enrolled patients with various types of cancer, including HCC [30], gastric cancer (GC) [31,33], PC [34], CRC [35], SCLC [36], and non-small-cell lung cancer (NSCLC) [32,37]. The used statin was pravastatin (40 mg/day) in three trials and simvastatin (40 mg/day) in five. Four studies adopted a randomized, double-blinded, placebo-controlled, clinical trial design [33,34,35,36]. The Jadad score was more than 3 in seven studies, indicating a good quality of the trials; one study [31] had a Jadad score of 2.

### 3.3. Impact of Statin Addition on Severe Adverse Events

**Figure 2:**
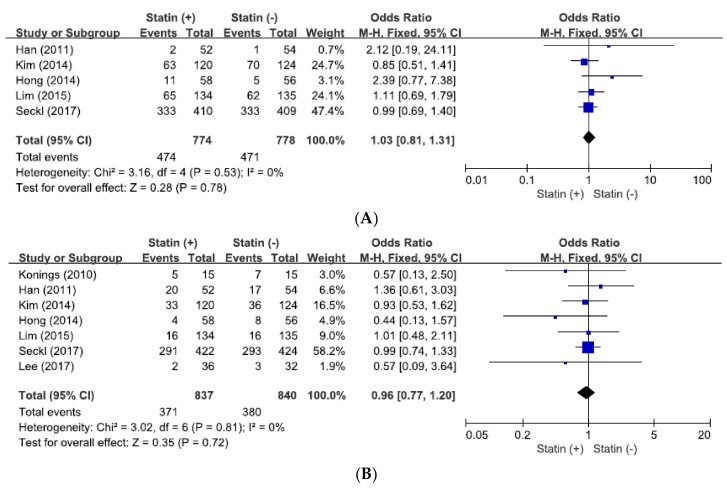
Forest plots for grade 3–5 adverse events (**A**) and overall response rate (**B**).

From five studies [32,33,34,35,36], a total of 1579 patients were included in combining ORs with 95% CIs for severe AEs. There was no significant heterogeneity among the studies (*X*^2^ = 3.16, *p =* 0.53, *I*^2^ = 0%) and the fixed-effect model was selected. Statins added to anticancer agents did not significantly increase the incidence of grade 3–5 AEs (OR = 1.03, 95% CI: 0.81–1.29, *p* = 0.78) (Figure 2A).

### 3.4. Effect of Statin Addition on Overall Response Rate

From seven studies [31,32,33,34,35,36,37], 1677 patients were included in pooling ORs with 95% CIs for ORR. There was no significant heterogeneity across the studies (*X*^2^ = 3.02, *p* = 0.81, *I*^2^ = 0%) and the fixed-effect model was used. The addition of statin to anticancer agents did not increase ORR (OR = 0.96, 95% CI: 0.77–1.20, *p* = 0.72) (Figure 2B).

### 3.5. Effect of Statin Addition on Progression-Free Survival

**Figure 3:**
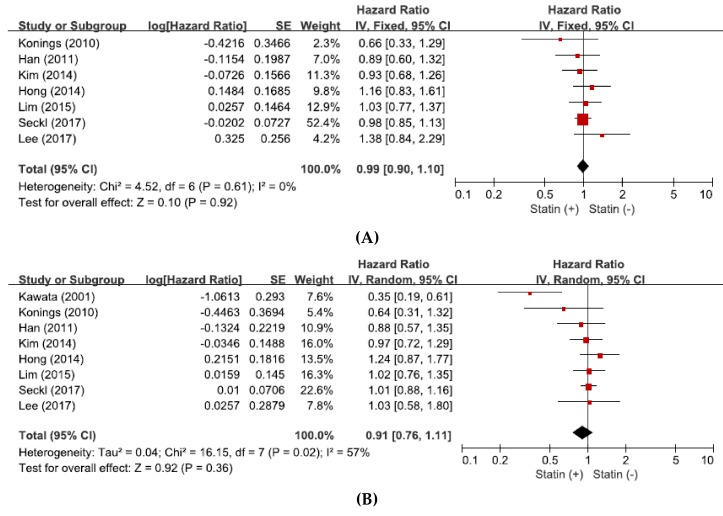
Forest plots for progression-free survival (**A**) and overall survival (**B**).

From seven studies [31,32,33,34,35,36,37], a total of 1677 patients were included in the pooled analysis of HRs with 95% CIs for PFS. Because there was no significant heterogeneity among the studies (*X*^2^ = 4.52, *p =* 0.61, *I*^2^ = 0%), the fixed-effect model was selected. The addition of statin to anticancer agents showed no significant impact on PFS (HR = 0.99, 95% CI: 0.90–1.10, *p* = 0.92) (Figure 3A).

### 3.6. Effect of Statin Addition on Overall Survival

From eight studies [30,31,32,33,34,35,36,37], a total of 1760 patients were included in the pooled analysis of HRs with 95% CIs for OS. There was a significant heterogeneity among the studies (*X*^2^ = 16.15, *p =* 0.02, *I^2^*= 57%) and the random-effects model was adopted. The addition of statin to anticancer therapy showed no significant impact on OS (HR = 0.91, 95% CI: 0.76–1.11, *p* = 0.36) (Figure 3B).

### 3.7. Publication Bias

**Figure 4:**
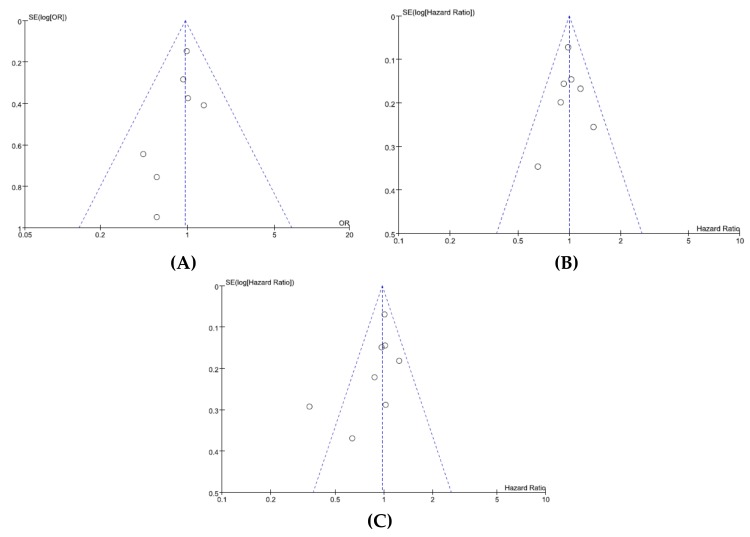
Funnel plots for publication bias: overall response rate (**A**); progression-free survival (**B**); overall survival (**C**).

We did not perform a publication bias test for grade 3–5 AEs because of a limited number of studies included. Visual inspection of the funnel plots for ORR (Figure 4A), PFS (Figure 4B), and OS (Figure 4C) indicated that there was no substantial publication bias. Egger’s tests also demonstrated the absence of significant publication biases (*p* = 0.198 for ORR, *p* = 0.991 for PFS, and *p* = 0.126 for OS, respectively).

## 4. Discussion

There has been growing interest in the effect of lipid-lowering agents among patients with cancer based on preclinical evidence of their antiproliferative, proapoptotic, anti-invasive, and radiosensitizing properties [12,13,14,15,16,44], This meta-analysis was conducted to assess the role of statins in the fight against cancer. The results indicated that the addition of satin to systemic anticancer therapy was not associated with improved clinical outcomes. To our knowledge, this is the first meta-analysis of randomized, controlled trials regarding the effects of statins added to systemic anticancer treatment.

Statins inhibit HMG-CoA reductase to lower mevalonic acid and its downstream products, many of which play important roles in critical cellular functions such as membrane integrity, cell signaling, protein synthesis, and cell cycle progression [11]. Therefore, perturbation of these processes by statins in cancer cells may result in control of tumor initiation, growth, and metastasis [45,46]. Preclinical studies have observed positive effects for statins in various cancer cell lines, including reduced proliferation and migration, increased apoptosis, and reduced tumor growth [12,13,14,15,16]. Many observational studies have also reported that stains can reduce recurrence or mortality in patients with various types of cancer [17,18,19,20,21,22]. In addition, a couple of phase II studies reported that statins in combination with chemotherapy was feasible and effective in patients with metastatic CRC [29] or extensive SCLC [47].

The first randomized controlled trial on the addition of statin to standard treatment was conducted in patients with HCC [30]. Eighty-three patients underwent transcatheter arterial embolization followed by oral 5-FU (200 mg/d) for 2 months. Patients were then randomly assigned to a control (*n* = 42) or pravastatin (40 mg/d) group (*n* = 41). Although this study was limited by a lack of blinding and small sample size, the results were promising. Median OS was 18 months in the pravastatin arm versus 9 months in control (*p* = 0.0006). The Cox proportional hazard model indicated pravastatin as a significant prognostic factor (HR = 0.35, 95% CI: 01.7–0.61, *p* = 0.005). Based on the promising results in retrospective studies or prospective phase II trials [29,30,47,48], seven randomized, controlled phase II or III trials in GC [31,33], PC [34], CRC [35], SCLC [36], and NSCLC [32,37] were published between 2010 and 2017. The statins (pravastain or simvastatin) was combined with cytotoxic chemotherapy or targeted therapy (gefitinib or afatinib). However, no studies indicated the addition of statin to anticancer agents to be more effective, compared with standard anticancer therapy alone.

This meta-analysis of the eight randomized, controlled trials revealed that the statins in combination with systemic anticancer therapy failed to draw any clinical benefits in patients with solid cancer. Statins as a group are generally well tolerated although muscle toxicity and asymptomatic liver enzyme elevation have been reported. In this meta-analysis, the addition of pravastatin or simvastatin to anticancer agents did not significantly increase the incidence of grade 3–5 AEs (OR = 1.03, 95% CI: 0.81–1.29, *p* = 0.78). However, the combination of statin and anticancer agents did not improve the radiological tumor response (OR of ORR = 0.96, 95% CI: 0.77–1.20, *p* = 0.72) compared with that of anticancer therapy alone. In agreement with the ORR result, statins added to systemic anticancer therapy failed to prolong PFS (HR = 0.99, 95% CI: 0.90–1.10, *p* = 0.92) and OS (HR = 0.91, 95% CI: 0.76–1.11, *p* = 0.52).

Several possibilities may explain the reasons why the addition of statins to anticancer agents failed to generate clinical benefits in patients with solid cancer. First, there might be actually no beneficial effects of statins added to anticancer agents in patients with solid cancer. There is no shortage of examples where compelling observational evidence of a drug effect fails to be demonstrated in randomized, controlled trials. A meta-analysis of 175,000 people from 27 randomized trials of statin therapy for cardiovascular prevention failed to show beneficial effects on the incidence of, or mortality from, any type of cancer [49]. The impressive effect of statins in reducing the incidence of and mortality from cancer might be associated with time-related biases (e.g., immortal time bias) in some observational studies [50]. Second, as we know, cancer is not a homogeneous disease entity. Therefore, the effects of statins may significantly differ according to anatomical site and molecular type of cancers. Of interest, a study in glioblstoma cells suggested that statins might fail to work in certain cancer cells because of a phosphatidylinostitol 3-kinase-mediated pathway connected to LDL receptors [51]. It is not known if this might affect the responsiveness to statins in other types of cancer. Third, the dose of statins adopted in the clinical trials might be insufficient. The mechanism of postulated anticancer effect depends on the inhibition of posttranslational processing via perturbation of the mevalonate pathway, the same mechanism involved in cholesterol synthesis. The statins in the included trials were administered at the same dosage as usually used in the treatment of hypercholesterolemia. However, the optimal dose of statins to display anticancer effects is not known. Although Lee at al. suggested an antitumor effect of simvastatin using a dose level that is equivalent to the accepted cardiovascular therapeutic dose level [29,52], other studies demonstrating anticancer effects have used high statin concentration that are not feasible for human use [53,54]. Fourth, there might be a particular group of patients who benefit more from the addition of statins to anticancer therapy. Lim et al. observed that simvastatin enhanced the antitumor activity of cetuximab in CRC cells carrying Kirsten-RAS (KRAS) mutations [35]. Statins may inhibit the expression of the mutant KRAS phenotype by preventing the prenylation of the KRAS protein and normalizing the phenotype into KRAS wild type and, therefore, rendering KRAS mutant CRCs sensitive to monoclonal antibodies at epidermal growth factor receptors [55]. Lim et al. hypothesized that statins might overcome cetuximab resistance in KRAS mutant CRC cells. However, statin use was not associated with improved PFS in patients treated with cetuximab for KRAS mutant metastatic CRC [56]. Another hypothesis is that anticancer effects of statins might be limited in advanced or metastatic settings with a large tumor burden. Many observational studies have reported that statin was associated with improved recurrence-free survival or reduced risk of death when used after curative resection of cancer [19,21,57]. However, a relatively small number of studies has focused on the effect of statins in advanced or metastatic settings [29,47]. In this meta-analysis, six studies were conducted in patients with advanced or metastatic cancer and no clinical benefits were observed [31,32,33,34,35,37].

This study has some inherent limitations. First, the small number of included studies is a major limitation of this meta-analysis. Thus, we could not perform subgroup analysis according to the primary site of cancers. Second, patients had different tumor types and received various therapeutic regimens in the different treatment settings (first-line or salvage setting). Third, there was a significant heterogeneity among the studies (*X*^2^ = 16.15, *p* = 0.02, *I*^2^ = 57%) when pooling HRs for OS, which was primarily associated with the study by Kawata et al. [30]. The heterogeneity could not be completely interpreted although the random-effects model was selected. Finally, articles published only in English were included, which might have lead to selection bias.

## 5. Conclusions

This meta-analysis of randomized, controlled, phase II or III trials does not support clinical benefits of statins added to systemic anticancer therapy in patients with solid cancer. However, further investigations are needed to resolve the issues (dose and type of statin, treatment setting, particular cancer type, or biomarkers) regarding the addition of statins to systemic anticancer therapy.

## Figures and Tables

**Table 1:** Eight randomized, controlled trials of systemic anticancer therapy with or without statin in human cancer.

First Author, (Year) [Ref.]	Cancer Type	Phase	Setting	Treatment Arm	No. of Patients	Primary Endpoint	ORR	Any Gr 3–5AEs	mPFS (Month)	mOS(Month)	Jadad Score
Kawata (2001) [30]	HCC	II	1st	TAE + oral 5-FU + pravastatin 40 mg	41	OS	NA	NA	NA	18	3
				TAE + oral 5-FU	42		NA	NA	NA	9	
Konings (2010) [31]	GC	II	1st	Epirubicin/cisplatin/capecitabine + pravastatin 40 mg	15	PFS	33.3%	8 (53.3%) *	6	8	2
				Epirubicin/cisplatin/capecitabine	15		46.7%	7 (46.7%) *	5	6	
Han (2011) [32]	NSCLC	III	2nd or 3rd	Gefitinib 250 mg + simvastain 40 mg	52	ORR	38.5%	2 (4%)	3.3	13.6	3
				Gefitinib 250 mg	54		31.5%	1 (2%)	1.9	12	
Kim (2014) [33]	GC	III	1st	Capecitabine/cisplatin + simvastatin 40 mg	120	PFS	27.5%	63 (52.5%)	5.2	11.6	5
				Capecitabine/cisplatin + placebo	124		29.0%	70 (56.4%)	4.6	11.5	
Hong (2014) [34]	PC	II	1st	Gemcitabine + simvastatin 40 mg	58	PFS	6.9%	11 (18.9%)	2.4	6.3	5
				Gemcitabine + placebo	56		14.3%	5 (9%)	3.6	8.7	
Lim (2015) [35]	CRC	III	2nd	XELIRI or FOLFIRI + simvastatin 40mg	134	PFS	11.9%	65 (48.5%)	5.9	15.3	5
				XELIRI or FOLFIRI + placebo	135		11.8%	62 (45.9%)	7.0	19.2	
Seckl (2017) [36]	SCLC	III	1st	Etoposide/platinum +/− RT + pravastatin 40 mg	422	OS	69.0%	333 (81.2%)	7.7	10.7	5
				Etoposide/platinum +/− RT + placebo	424		69.1%	333 (81.4%)	7.3	10.6	
Lee (2017) [37]	Non-ADC NSCLC	II	2nd or 3rd	Afatinib + simvastatin 40 mg	36	ORR	5.7%	2 (5.6%) ^‡^	1.0	10	3
				Afatinib	32		9.4%	6 (16.8%) ^‡^	3.6	7	

HCC, hepatocellular carcinoma; GC, gastric cancer; NSCLC, non-small-cell lung cancer; PC, pancreatic cancer; CRC, colorectal cancer; SCLC, small-cell lung cancer, non-ADC, non-adenocarcinoma; TAE, transcatheter arterial embolization; 5-FU, 5-fluorouracil; XELIRI, capecitabine + irinotecan; FOLFIRI, 5-FU + leucovorin + irinotecan; RT, radiotherapy; AEs, adverse events; ORR, overall response rate; mOS, median overall survival; mPFS, median progression-free survival; OR, odds ratio; HR, hazard ratio; NA, not available. CI, confidence interval; * neutropenia, ^‡^ diarrhea.

## Abbreviations

CI	confidence interval
CRC	colorectal cancer
GC	gastric cancer
HCC	hepatocellular carcinoma
HMG-CoA	3-hydroxy-3-methylglutaryl-coenzyme A
HR	hazard ratio
LDL	low-density lipoprotein
Non-ADC	non-adenoarcinomatous
NSCLC	non-small-cell lung cancer
PC	pancreatic cancer
PFS	progression-free survival
OR	odds ratio
OS	overall survival
SCLC	small-cell lung cancer
